# Feasibility and Safety of ERCP in the Treatment of Biliary Strictures after Liver Transplantation: With a Report of 37 Cases

**DOI:** 10.1155/2022/4498443

**Published:** 2022-08-23

**Authors:** Fanfan Tang, Jingtao Song, Tanxing Cai, Zhao Lei, Feizhou Huang, Yina Hu, Gang Deng

**Affiliations:** ^1^Wuhan Children's Hospital (Wuhan Maternal and Child Healthcare Hospital), Tongji Medical College, Huazhong University of Science & Technology, Wuhan, Hubei 430015, China; ^2^Department of Hepatobiliary Surgery, The Third Xiangya Hospital of Central South University, Institute of Hepatobiliary and Pancreatic Minimally Invasive Surgery, Central South University, Changsha, Hunan 410013, China; ^3^Women and Children's Hospital of Hunan, Changsha, Hunan 410000, China; ^4^Wuhan Donghu University, Wuhan, Hubei 430212, China

## Abstract

**Background:**

Liver transplantation (LT) is an effective treatment option for patients with end-stage liver disease; biliary complications are important cause of death in posttransplant patients. Endoscopic retrograde cholangiopancreatography (ERCP) has an irreplaceable role in the diagnosis and treatment of patients with biliary tract disease.

**Methods:**

The clinical data of patients with biliary strictures (BS) after LT treated with ERCP admitted to the Third Xiangya Hospital of Central South University from September 2016 to October 2021 were reviewed; the changes in temperature, bilirubin, and albumin before and after treatment and postoperative complications were analyzed.

**Results:**

A total of 41 patients were included in the study, and biliary stents were successfully placed in 37 cases (90.2%), while 4 cases (9.8%) were unsuccessful due to complete BS. Patients with ERCP guided biliary stenting had a significant improvement in bilirubin index compared to the preoperative period (*P* < 0.05). 27 patients (73.0%) had complete relief of symptoms after 1 ERCP-guided treatment, and 10 patients (27.0%) developed BS again at different times after the first ERCP treatment, among which 8 patients developed BS again within 1 year after the first treatment and 2 patients developed BS again after 1 year after the first treatment. The incidence of endoscopy-related adverse events was 35.14%, with no serious adverse events.

**Conclusion:**

ERCP-guided biliary stenting was an effective and safety treatment for BS after LT.

## 1. Introduction

Liver transplantation (LT) is an effective treatment option for patients with end-stage liver disease. Patients are generally less able to resist disease after LT, and biliary complications still threaten the life and health of posttransplant patients and constitute an important cause of morbidity in patients after LT [1, 2]. A related study noted that biliary-related complications occurred in approximately 23% of patients after LT, with the most common complication being biliary stricture (BS) (14.7%) [3]. BS can increase the pressure of the biliary duct and obstruct bile excretion, causing retrograde bacterial infection and further aggravating liver injury. Some patients may suffer from jaundice, itchy skin, and digestive mild symptoms. In severe cases, systemic inflammatory response syndrome, multiple organ dysfunction syndrome, shock, and even liver failure of varying degrees may occur, seriously affecting the quality of life [4, 5]. Timely and effective management of these complications to prevent irreversible liver injury and improve the quality of life of patients is particularly important.

Posttransplant patients with pain, abnormal liver function, fever, and bile draining from the abdominal cavity need to be examined to determine the presence of BS. Cholangiography is one of the criteria for determining BS, and endoscopic retrograde cholangiopancreatography (ERCP) is a microinvasive technique that uses endoscopy and X-ray technology to examine and treat pancreatic and biliary diseases, with both diagnostic and therapeutic functions. Giovannini et al. [6] concluded that endoscopy allows both clear visualization of biliary structures and access to the biliary tree through the gastrointestinal tract on the basis of less-invasive operations. Several studies have recognized the effectiveness of ERCP in BS after LT. Álvarez et al. [7] showed that ERCP can be 92.3% effective in the treatment of BS and that patients had a low rate of ERCP complications with a mild degree. Girard et al. [8] concluded that internal biliary stenting can decrease biliary complications after LT. Erdoğan et al. [9] also showed that ERCP is more than 90% effective in the treatment of patients with BS after live donor LT, and its advantages of comprehensiveness, effectiveness, and safety make it irreplaceable in the diagnosis and treatment of patients with biliary diseases.

The most common complications in patients after ERCP are pancreatic hyperamylasemia, intestinal perforation, and biliary fistula, which may affect the expected outcome of ERCP. In studies on ERCP for BS after LT, the efficiency rate is above 80% [9, 10]. However, the effectiveness of ERCP is influenced by the operator's technique, the patient's economic situation, the degree of BS, timely access to medical care, patient compliance, and sample size. In this study, we investigated the clinical efficacy and safety of ERCP in patients with BS after LT by retrospectively analyzing the clinical data of patients who underwent ERCP for BS after LT in our center in the past 5 years.

## 2. Methods

### 2.1. Study Subjects

Patients with BS after LT treated with ERCP-guided biliary stent admitted to the Third Xiangya Hospital of Central South University from September 2016 to October 2021 were selected for the study, and all patients were excluded from graft rejection or bile duct ischemia before ERCP treatment. The following are the inclusion criteria: (1) age ranged from 18 to 70 years; (2) diagnosis by imaging-related examinations (CT/MRCP/cholangiography) or endoscopy according to the diagnostic guidelines for BS after LT, meeting the diagnostic criteria for BS; (3) indications for endoscopic treatment; (4) significant changes in patients' symptoms and signs, with significant yellow staining of the sclera and general skin, significant liver function abnormalities, and significantly elevated white blood cell count; and (5) complete and reliable clinical data for postoperative follow-up. The following are the exclusion criteria: (1) patients with severe cardiovascular diseases, such as myocardial infarction, coronary artery disease, cerebral infarction, and cerebral hemorrhage; (2) patients with pregnancy and hepatic artery stricture/thrombosis; (3) BS caused by benign or malignant tumors of the biliary tract, compression by gastrointestinal masses, congenital anomalies of the biliary tract, and iatrogenic factors; (4) long-term use of liver-damaging drugs and history of drug abuse; (5) patients with other prevalent susceptible diseases; and (6) patients with dementia, mental abnormalities, and other cognitive impairments that make it difficult to cooperate with the follow-up. A total of 41 patients with BS after LT who met the criteria were included, 37 cases were successfully placed with biliary stents, while 4 cases (9.8%) were unsuccessful due to complete BS, and a total of 37 study subjects were finally included. All patients signed informed consent forms, and all operations were performed within the scope of clinical safety.

### 2.2. Equipment and Operation Procedure

The following are the equipment used: mainframe, electronic gastroscopy, plastic stent (PS) (Boston Scientific, USA), metal stent (MS) (Boston Scientific, USA), invasive balloon dilator (BD) (Nanjing Minimally Invasive Medical Technology Co., Ltd.), Boston zebra guide wire, stone extractor, and nasobiliary tube. The materials of MS and PS are nickel-titanium alloy and Teflon, respectively.

**Figure 1:**
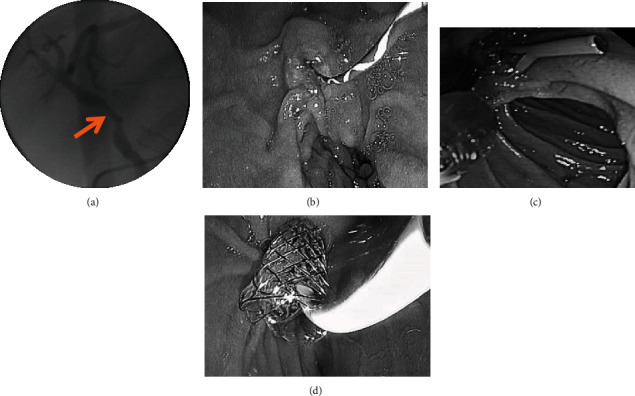
Surgical procedure of ERCP in the treatment of biliary strictures after liver transplantation. (a) Before ERCP, cholangiography showed biliary strictures; (b) the Boston zebra guide wire was placed into the intrahepatic bile duct; (c) the plastic stent was successfully placed with balloon dilator; (d) metal stent placed successfully.

For the operation procedure, the patient was placed in the left recumbent position, with lidocaine mucilage sublingual or intravenous propofol, and the operation was performed under the direct view of the electronic gastroscopy. The examination process was performed by skilled endoscopists in our hospital, and the treatment was decided by the endoscopists. During the treatment, the patient's vital signs were monitored, and the site and degree of BS, the length of the strictured segment, the presence of surrounding benign and malignant masses for compression, and the patency of the biliary were observed in detail, and the patient was assessed for the need of endoscopic sphincteropapillotomy (EST) according to the situation. The Boston zebra guide wire was placed into the intrahepatic bile duct in the direction of the bile duct, and depending on the degree of strictures, the BD was added to expand the narrow segment of BS, and then a specific stent (plastic/metal) or nasobiliary tube was placed into the biliary in the direction of the guide wire. Relevant pictures are shown in Figure 1.

After the operation, we observed the condition of the drainage tube, including the nature, color, and amount of fluid drainage, and if necessary, appropriate flushing was given to keep the drainage tube unobstructed. The liver function was rechecked 3 and 7 days after surgery, and the changes of bilirubin, albumin, and other indexes were recorded in detail. During the postoperative treatment, the patient's clinical symptoms, cholangiography results, and changes in fluid drainage were comprehensively evaluated. If the patient's clinical symptoms do not improve significantly, the obstruction site was still not released or further aggravated by rechecked cholangiography, the drainage tube was blocked or other causes of poor drainage, further endoscopic treatment can be performed, and finally, the biliary stent can be removed according to the patient's clinical and imaging performance as appropriate.

### 2.3. Definitions

- BS: cholangiography showed BS, ineffective passage, or poor flow of contrast medium.
- Surgical success: successful biliary stent placement under ERCP and cholangiography showed the patency of biliary.
- Stricture resolution: stricture resolution was determined by the physician according to the cholangiography, or at the end of endoscopic treatment, the 8.0 mm balloon easily passed through the anastomosis, the contrast medium can be emptied rapidly, and biochemical indices about the liver blood and clinical symptoms were improved.
- Recurrent biliary obstruction (RBO): there is a recurrence of clinical symptoms (hyperthermia, epigastric pain) and/or increased bilirubin, requiring follow-up intervention.

### 2.4. Follow-Up

Patients were followed up from the time of death and the last follow-up visit as the cut-off date (>15 days were calculated according to 1 month), and the follow-up period date for patients was up to November 30, 2021, mainly to observe the patients' disease recurrence and quality of survival.

### 2.5. Quality Control

During the study period, all procedures were performed by two experienced biliary endoscopists at our institution. During the data collection phase, the data were coded and analyzed by two researchers, and missing values were assigned using the mean value method.

### 2.6. Observation Indexes

The following are the observation indexes:

1. Biochemical indicators: these include changes in temperature, bilirubin, albumin, and white blood cell count (WBC) of patients before and after treatment.
2. Treatment effect: Analysis of patient treatment effect and recurrent biliary obstruction. For cured or improved, the patients' clinical symptoms completely disappeared or were significantly relieved, the biochemical indexes returned to normal, the cholangiography indicated that the BS had been released or largely resolved, and no complications occurred or the complications were cured after surgery; for invalid, the clinical symptoms did not improve or worsened, with unsuccessful placement of biliary stents and poor treatment results after surgery.
3. Adverse events or complications related to endoscopic treatment: these include stent displacement, post-operative pancreatic hyperamylasemia, intestinal perforation, bleeding, and gastrointestinal fistula.
4. Time of stent placement: this is the time period from stent placement to final removal.
5. Time to recurrent biliary stricture: this is the interval between initial placement and recurrence of obstruction. If patients did not experience recurrent biliary obstruction, the date of death or the follow-up time cutoff was used as the end point of the follow-up.

### 2.7. Statistical Analysis

All statistical analyses were performed using IBM SPSS Statistics 18.0 software (SPSS Inc., Chicago, IL, USA). The measurement data were normally distributed and described by mean and standard deviation, and comparisons between different time periods were analyzed by repeated measures ANOVA; if the data did not conform to a normal distribution, the median (*P*_25_, *P*_75_) was used to describe, and the Friedman *M* test and Wilcoxon test were used. Count data were expressed as cases and percentages, using a chi-square test or Fisher's exact test. The Kaplan-Meier test was used to nonobstruction rates after stent placement at 3, 6, and 12 months estimated. All *P* values were two-sided. Differences were considered significant for *P* < 0.05.

## 3. Results

### 3.1. Patient Characteristics

Because of 4 cases of complete BS, 37 study subjects were finally included in this study. Among the study subjects, 33 cases (89.2%) were male and 4 cases (10.8%) were female, aged 46.54 ± 10.05 years, and there were 8 cases (21.6%) (≤39 years old). The time from transplantation to the first BS in the patients ranged from 8 to 730 days, with a mean of 106 days and a median of 75 days (*P*_25_ = 40 d, *P*_75_ = 131 d); all study subjects were treated with antibiotics postoperatively. The characteristics and clinical details of the study subjects are shown in Table 1.

### 3.2. Comparison of Temperature and Laboratory Parameters of Patients before and after Treatment

The patients' temperature before surgery ranged from 36.00°C to 39.60°C, with a median of 37.0°C (*P*_25_ = 36.7°C, *P*_75_ = 37.9°C), and postoperative temperature ranged from 36.00°C to 38.90°C, with a median of 37.2°C (*P*_25_ = 36.7°C, *P*_75_ = 37.6°C). The difference was not statistically significant in temperature before and after operation (*Z* = −0134, *P* = 0.893). The differences in WBC, total bilirubin (TBIL) and direct bilirubin (DBIL) were statistically significant when comparing before operation, 3 days and 7 days after operation, as shown in Table 2. A pair-wise comparison is shown in Table 3.

### 3.3. Albumin

One-way repeated measures ANOVA was used to determine the changes in albumin. The data conformed to normal distribution by Shapiro-Wilk test (*P* > 0.05), and the variance covariance matrix of the dependent variable was equal by Mauchly's test of sphericity, chi-square test = 3.881, and *P* = 0.144. Albumin data were expressed as the mean ± standard deviation, with 36.74 ± 4.97 g/L at preoperative, 36.32 ± 4.79 g/L at 3 days postoperative, and 36.87 ± 3.78 g/L at 7 days postoperative. The difference in albumin before and after operation was not statistically significant (*F* = 0.364, *P* = 0.696, partial Eta-squared = 0.010).

### 3.4. Clinical Efficacy

Follow-up results showed that 27 patients (73.0%) had gradual relief of symptoms after 1 ERCP treatment, and no recurrence of BS occurred during the follow-up period. Compared with before treatment, bilirubin significantly decreased to normal level in 24 patients, and there was an upward trend in 3 patients after ERCP treatment but decreased to normal level within 2 weeks. 10 patients (27.0%) had gradual relief of symptoms after 1 ERCP treatment, but developed RBO during the follow-up period. Eight of them developed RBO within 1 year after the first treatment, and two of them developed RBO after 1 year after the first treatment. No deaths or serious adverse complications as of the follow-up time.

### 3.5. Stricture Management

The number of ERCP treatments for patients was 1.43 ± 0.80. After the patients developed first stricture, MS were placed in 11 cases (29.7%) and PS were placed in 26 cases (70.3%). Among the patients with MS placed, 8 (21.6%) had MS placed after expansion by dilatation strips, 1 (2.7%) had MS placed after BD, and 2 (5.4%) had MS placed directly without expansion. Of the patients with PS, 9 (24.3%) had endoscopic nasobiliary drainage (ENBD), 6 (16.2%) had ENBD after expansion by dilatation strips, 1 (2.7%) had plastic stent placed after expansion by dilatation strips, 3 (8.1%) had ENBD after BD, and 7 (18.9%) had PS placed only.

### 3.6. Management of Patients Presenting with Recurrent Strictures

Ten patients developed recurrent BS (second BS after postliver transplantation) after the first ERCP treatment. Among these patients, the time interval between initial placement and recurrence of obstruction was less than 6 months in 8 cases and more than 1 year in 2 cases. In terms of treatment, MS were placed in 3 cases (30.0%), including 1 case after BD, 1 case with MS alone, and 1 case after expansion by dilatation strips. PS were placed in 7 cases (70.0%), including ENBD in 1 case, PS after BD in 2 cases, ENBD after BD in 1 case, and PS alone in 3 cases.

After the 2nd ERCP treatment, 5 patients (50.0%) showed gradual clinical symptom relief, and 5 patients (50.0%) developed a third BS, with an interval of within 6 months after the second treatment in 1 case, 7~12 months in 1 case, and more than 12 months in 3 cases. In terms of treatment, 2 cases with ENBD after BD, 1 case with PS, 1 case with PS after expansion by dilatation strips, and 1 case with PS after BD. One patient developed a fourth BS one year later, and a PS was placed after BD to manage the BS.

### 3.7. Total Stent Placement Time and Time to Recurrent Biliary Obstruction

As of the follow-up time, the total stent placement time for the study subjects was 137.22 ± 104.00 days. For patients with one stricture, the stent placement time ranged from 14 to 223 days, with a median placement time of 80 days and a mean of 97.11 ± 55.45 days; for patients with two strictures, the stent placement time ranged from 96 to 356 days, with a median placement time of 155 days and a mean of 177.00 ± 112.02 days; for patients with three strictures, the stent placement time ranged from 184 to 432 days with a median placement of 285.5 days and a mean of 296.75 ± 120.97 days. The duration of stent placement in patients with four strictures was 383 days. Up to the follow-up time, four patients had incomplete resolution of BS, two patients had PS placed, and two patients had MS placed.

Due to the long follow-up period of this study, in order to exclude missing or incorrect data due to patients not being treated at our hospital at a later time or lost visits, only the time of recurrent biliary obstruction in patients with RBO was analyzed in this study. There are 9 cases whose first stent placement type was PS and 1 patient whose first stent placement type was MS developed RBO, for the cause of obstruction: 1 case was stent displacement, 9 cases were stent obstruction due to gallstone, and the time of RBO ranged from 32 to 194 days, with a median of 43 days in the PS group (*n* = 9) (*P*_25_ = 33.5, *P*_75_ = 107.5) and 176 days in the MS group (*n* = 1). For all study subjects, the recurrent biliary stricture-free rates at 3 months, 6 months, and 1 year were 80.5%, 73.6%, and 70.1%, respectively, and the nonobstruction rates after initial placement at 3 months, 6 months, and 1 year were 72.9%, 68.0%, and 62.8% in the PS group and 88.9% in both MS groups, respectively (Log‐rank = 2.421, *P* = 0.120).

### 3.8. Occurrence of Endoscopy-Related Adverse Events

After the first ERCP procedure in 37 patients, 10 cases developed pancreatic hyperamylasemia, 1 case developed choledochoduodenal fistula, 1 case developed biliary fistula, and 1 patient developed stent dislodgement, and no serious cholangitis or sepsis occurred. The average treatment time of pancreatic hyperamylasemia was 3.56 ± 1.74 days, among which 2~3 days for patients with mild pancreatic hyperamylasemia and 5~7 days for patients with severe pancreatic hyperamylasemia.

### 3.9. Effect of the Type of Stent First Placed and EST on RBO and Adverse Events

Stent type had no effect on stricture recurrence rate or adverse event rate, as shown in Table 4. Six of the patients who underwent EST had complications, and the difference was statistically significant compared with those who did not undergo EST, as shown in Table 5.

## 4. Discussion

### 4.1. Effectiveness and Safety of ERCP in BS in Patients after LT

Biliary stricture is a common postoperative complication in LT patients and is one of the most important reasons for reducing the quality and survival of patients. BS usually occurs within 6 months after surgery and often causes jaundice and elevated liver enzyme levels in patients. ERCP or magnetic resonance cholangiopancreatography (MRCP) can diagnose BS, both of which have high sensitivity and specificity (>90%) [11]. MRCP is noninvasive, but its availability is limited, whereas ERCP has the advantage of integrating diagnosis and treatment. Compared to surgery, ERCP is less invasive and is one of the main tools for treating patients with BS after LT. The success rate of ERCP for BS can reach more than 70% when anatomical conditions allow [12, 13]. A standardized and prospective observational study showed that 53 patients with anastomotic stricture after LT can achieve 69% success rate after endoscopic treatment, with most patients requiring an average of three ERCP procedures and two 24F BD, and a median continuous stent retention time of 11 months [14]. Another study showed that patients with anastomotic strictures undergoing ERCP achieved clinical success in 92.3% [7]. It was also found that patients with biliary complications who underwent ERCP had a 1-year survival rate of 90% [15]. This indicates that patients with biliary complications after LT who underwent ERCP had higher overall survival and graft survival than those who did not undergo ERCP. In this study, patients with one stricture had stent placement from 14 to 223 days, with a median placement time of 80 days and a mean of 97.11 ± 55.45 days, 89.2% of patients had postoperative BS appearing within 6 months, 27 patients (73.0%) had complete relief of symptoms after one ERCP treatment, and bilirubin eventually decreased to normal levels, indicating that ERCP is efficient and safe for the treatment of BS complicating patients after LT.

### 4.2. Effect of ERCP on Complications in Patients after LT

ERCP can effectively treat BS by placing stents, removing biliary stone, and treating biliary leaks and cholangitis. However, ERCP is still an invasive procedure that may cause complications and reduce the safety and effectiveness of treatment. Hüsing et al. [16] analyzed the complication rate of 157 LT recipients within 15 days after endoscopic sphincterotomy and showed a complication rate of 15.3% (24/157), including 9 cases of pancreatic hyperamylasemia (5.7%), and most of the complications could be resolved with conservative treatment, which was considered a reasonable rate of surgery-related complications. The results of this study showed that the postoperative incidence of patients (excluding stent dislodgement) was 32.43%, which was similar to the results of complications reported by Álvarez et al. [7] (30.1%), but higher than the studies by Egea-Valenzuela et al. [17] and Balderramo et al. [18], which may be due to the small sample size of this study; the older age of the patients at the time of transplantation and EST was performed. The purpose of EST is the enlargement of the biliary opening to allow access to the instruments for the associated treatment, but excessive electrocoagulation of the papillary opening during the operation may damage the pancreatic tissue and lead to pancreatic hyperamylasemia in patients. Therefore, the need for EST during ERCP is controversial. The European Society of Gastrointestinal Endoscopy Guidelines state that EST is a risk factor for patients developing pancreatic hyperamylasemia [19]. However, two meta-analyses have shown that early precut sphincterotomy does not increase the risk of pancreatic hyperamylasemia for patients with difficult biliary access [20, 21]. The results in Table 5 show that EST had an effect on the rate of complications in patients, but most of the patients' post-ERCP complications were short-term, and those who developed complications were successfully cured after 2 to 7 days of effective treatment without serious adverse effects, indicating that EST during ERCP is a safe procedure for patients with BS in LT. Thiruvengadam et al. [22] noted that rectal administration of indomethacin after ERCP significantly reduced the incidence of pancreatic hyperamylasemia and that tacrolimus further reduced the risk in patients, and the use of both drugs prevented the development of moderate or severe pancreatic hyperamylasemia. The European Society of Gastrointestinal Endoscopy also recommends routine rectal administration of diclofenac or indomethacin 100 mg NSAIDs before ERCP in patients without contraindication [19]. In view of the complication rate in this study, further improvement of the technical level of the endoscopist and timely and effective preoperative and postoperative medication may effectively reduce the incidence of complications in patients after ERCP.

### 4.3. Effect of the Type of the First Stent Placement on Patient Treatment during ERCP

One study showed that the success rate of BD combined with stent placement was higher than that of BD alone in 25 post-LT patients who developed BS after LT [23]. BD is an effective noninvasive technique, and the combination of PS has led to a significant improvement in the endoscopic treatment of patients with BS after LT [24]. PS is the preferred method for patient of benign BS, and more studies have been conducted on the treatment of BS with metal stents; however, due to inconsistent reports on efficacy, cost, and complications, the choice of metal stents should be made on a case-by-case basis [25, 26]. Some studies have pointed out that for benign BS, plastic stents can be chosen first, and if the doctor judges that the stricture cannot be resolved in a short time, metal stents can be chosen depending on the situation [27, 28]. It has been pointed out that for patients with malignant biliary obstruction, there is no statistically significant difference between the MS group and the PS group in terms of clinical placement success rate, adverse events, median patency, and survival, but the cost effectiveness of PS is higher than that of MS [29]. PS is simple to operate, inexpensive, and easy to replace, but the biggest disadvantage is that multiple ERCP procedures are required to replace the stents to complete the treatment, as PS have to be replaced approximately every 3 months, with the number and diameter of stents increasing until the stricture disappears. MS can improve the rate of nonobstruction, but the main disadvantage of MS is that it needs to cross the normal distal biliary to reach the stricture, increasing the risk of stent migration or secondary biliary injury. The study noted that the incidence of adverse events in patients with MS and PS placed during ERCP procedures was 23.3% and 6.4%, respectively, and the incidence of acute pancreatic hyperamylasemia was 13.3% in the MS group and 2.1% in the PS group [30]. Martins et al. [30] showed a higher rate of stricture recurrence in the fully covered self-expandable metal stents (cSEMSs) group compared with the PS group, and the authors suggested that it may be the shorter residence time of cSEMSs in the biliary (6 months) in their study, which may be the reason for the higher recurrence rate in the MS group compared to the PS group (residence time of 12 months). Martins et al. [30] also found that six of the eight patients who had RBO after treatment with MS recovered successfully after a new round of treatment (placement of PS). A meta-analysis showed that MS had advantages in terms of number of ERCP procedures, treatment time, number of stents placed, and cost but were not statistically significant compared with PS in terms of stricture recurrence rates and adverse event rates [31]. A study concluded that cSEMSs did not have an advantage over PS in terms of strictures resolution rates and adverse event rates [24]. Another study found postoperative complications of 10% and 50% in patients with BS after LT using cSEMSs versus PS, respectively, but the difference in complication rates was not statistically significant (*P* = 0.051) [32]. In this study, one case of RBO was observed in patients with MS, and nine cases of RBO were observed in patients with PS; the proportion of RBO was 34.6% and 9.1% in patients with plastic and metal stents, respectively. However, the differences were not statistically significant when comparing the rates of RBO and adverse events. It indicates that both MS and PS have the benefits in ERCP. RBO is related to the primary disease and disease progression, and the most appropriate stent should be selected according to the specific situation in the procedure and the economic condition of the patient. Biodegradable stents and membrane-covered biliary stent have also emerged, but they are not widely used due to the relatively high price. In this study, more than 70% of patients chose PS for the first placement, possibly may be related to the fact that PS are less expensive. Both metal and plastic stents have relative merits, and there is still a lack of clear consensus regarding the preference between them. In general, from the perspective of clinical success rate and risk of complications, PS is currently a good choice for patients with benign BS, MS may be an economical and effective treatment for patients with benign BS, but its role remains to be further elucidated. The selection of stents during surgery may be based on patient survival, physician expertise, and consideration of patient claims and cost-effectiveness, especially for low-income and uninsured patients, liver transplantation already imposes a significant financial burden on the family, and for whom cost may be more of a consideration in the selection of stents. Therefore, multicenter, prospective randomized controlled studies are needed to further explore the selection of biliary stents in endoscopic treatment.

### 4.4. Impact of ERCP on Patients' Laboratory Indicators

Laboratory indicators are important information reflecting the condition, and timely follow-up of changes in the indicators provides insight into changes in the patient's condition. The aim of BS treatment is to normalize the flow of bile, and there is a relationship between the resolution of BS and laboratory indicators. There is a study that has pointed out that TBIL is significantly higher in patients with BS after LT than in patients without strictures [33], suggesting the correlation between bilirubin concentration and the development of BS. Al-Mofleh et al. [34] concluded that bilirubin levels are the best predictor of malignant BS, the predictive sensitivity of malignant BS was 98.6%, and the specificity was 59.3% when the serum bilirubin level was greater than 84 *μ*mol/L and above. It has also been noted that the serum bilirubin trough in the first 30 days was significantly lower in patients with BS after LT who survived within one year (0.86 mg/dL) than in those who did not (1.07 mg/dL, *P* = 0.043) [35]. Higher WBC and TBIL and lower serum albumin levels were associated with cholangitis due to stent obstruction after ERCP [36]. The results of this study showed that after first ERCP, patients showed a decreasing trend of TBIL and DBIL, the WBC 7 days after surgery was lower than the preoperative, and the differences were all statistically significant (*P* < 0.05). It shows that ERCP can effectively relieve BS and reduce the inflammatory response of the body, which is an effective method for treating BS. In our study, there was no statistically significant difference in albumin counts before and after treatment, which may be related to the patients' timely access to medical care after the onset, the current implementation of enhanced recovery after surgery, and early nutritional support.

## 5. Limitations

The shortcomings of this study are that the study was retrospective with a small sample size and did not accurately answer questions such as the best stenting protocol and stent duration. In addition, the choice of stent was determined by the physician after discussion with the patient and was influenced by many factors, such as the patient's own disease condition, financial condition, and trust in the physician. In addition, some patients may be seen at other hospitals during treatment, and the medical records of ERCP treatment were not well documented; patient data were not effectively shared among clinical centers, which may have affected the accuracy of ERCP treatments in this study.

## 6. Conclusion

In conclusion, endoscopic stent placement is an effective treatment for most biliary complications, early diagnosis, and aggressive treatment are essential to reduce morbidity and mortality in patients with biliary complications after LT.

## Figures and Tables

**Table 1:** The characteristics and clinical details of study subjects.

Items	*n* (%)
Gender	
Male	33 (89.2)
Female	4 (10.8)
Age (years)	46.54 ± 10.05
18~39	8 (21.6)
40~59	26 (70.3)
60~70	3 (8.1)
Type of pretransplant disease	
Liver failure	7 (18.9)
Cirrhotic decompensation	16 (43.2)
Alcoholic liver disease	4 (10.8)
Liver cancer	7 (18.9)
Others	3 (8.1)
BMI (kg/m^2^)	19.20 ± 2.01
<18.5	20 (54.1)
18.5~24.0	16 (43.2)
>24.0	1 (2.7)
Chronic disease conditions	
Yes	17 (45.9)
No	20 (54.1)
Type of first ERCP stent placement	
Metal stent	11 (29.7)
Plastic stent	26 (70.3)
Time to first stricture after LT (months)	
<3	22 (59.5)
3~	11 (29.7)
6~	2 (5.4)
12~	2 (5.4)
EST	
Yes	7 (18.9)
No	30 (81.1)

**Table 2:** The comparison of WBC, TBIL, and DBIL.

Items	Preoperative	3 days postoperative	7 days postoperative	Friedman *M*	*P* value
WBC: (×10^9^/L)	7.90 (4.77, 9.28)	5.95 (4.77, 7.95)	6.24 (4.60, 7.12)	7.946	0.019
TBIL: *μ*mol/L	113.90 (60.35, 154.65)	54.90 (37.80, 86.55)	35.66 (23.58, 67.08)	42.541	<0.001
DBIL: *μ*mol/L	82.50 (41.25, 119.85)	37.60 (19.20, 62.60)	25.10 (12.70, 44.34)	45.622	<0.001

**Table 3:** The pair-wise comparison of WBC, TBIL, and DBIL.

Comparison group	*Z* ^∗^ value	*P* value
WBC		
3 days postoperative vs. preoperative	-1.079	0.281
7 days postoperative vs. preoperative	-2.678	0.007
7 days postoperative vs. 3 days postoperative	-1.727	0.084
TBIL		
3 days postoperative vs. preoperative	-4.247	<0.001
7 days postoperative vs. preoperative	-4.337	<0.001
7 days postoperative vs. 3 days postoperative	-4.081	<0.001
DBIL		
3 days postoperative vs. preoperative	-4.232	<0.001
7 days postoperative vs. preoperative	-4.458	<0.001
7 days postoperative vs. 3 days postoperative	-4.271	<0.001

^∗^Wilcoxon signed-rank test.

**Table 4:** Effect of MS and PS on RBO and adverse events.

Groups	RBO		Adverse events	
	Yes	No	Yes	No
MS	1 (9.1)	10 (90.9)	6 (54.5)	5 (45.5)
PS	9 (34.6)	17 (65.4)	7 (26.9)	19 (73.1)
*P* ^∗^	0.224		0.143	

*P* ^∗^: Fisher's exact test.

**Table 5:** Effect of EST on RBO and adverse events.

EST	RBO		Adverse events	
	Yes	No	Yes	No
Yes	0 (0.0)	7 (100.0)	6 (85.7)	1 (14.3)
No	10 (33.3)	20 (66.7)	7 (23.3)	23 (76.7)
*P* ^∗^	0.155		0.004	

*P* ^∗^: Fisher's exact test.

## Data Availability

All data generated and analyzed in this study were included in this published article.

## Abbreviations

ERCP: Endoscopic retrograde cholangiopancreatography
LT: Liver transplantation
BS: Biliary strictures
PS: Plastic stents
MS: Metal stents
BD: Balloon dilation
EST: Endoscopic sphincteropapillotomy
ENBD: Endoscopic nasobiliary drainage
RBO: Recurrent biliary obstruction
WBC: White blood cell count
TBIL: Total bilirubin
DBIL: Direct bilirubin
cSEMSs: Covered self-expandable metal stents.
